# Utility of visual rating scales in primary progressive aphasia

**DOI:** 10.1186/s13195-024-01442-7

**Published:** 2024-04-06

**Authors:** Neus Falgàs, Luca Sacchi, Tiziana Carandini, Nuria Montagut, Giorgio Conte, Fabio Triulzi, Daniela Galimberti, Andrea Arighi, Raquel Sanchez-Valle, Giorgio Giulio Fumagalli

**Affiliations:** 1 Alzheimer’s Disease and Other Cognitive Disorders Unit, Neurology Service, Hospital Clínic de Barcelona, FRCB Institut d’Investigacions Biomèdiques August Pi i Sunyer (IDIBAPS), Universitat de Barcelona, Barcelona, Spain; 2 Department of Biomedical, Surgical and Dental Sciences, University of Milan, Milan, Italy; 3 Neurodegenerative Diseases Unit, Ospedale Maggiore Policlinico, Fondazione IRCCS Ca’ Granda, Milan, Italy; 4 Department of Pathophysiology and Transplantation, University of Milan, Milan, Italy; 5 Center for Mind/Brain Sciences (CIMeC), University of Trento, Corso Bettini 31, Rovereto, 38068 Italy

**Keywords:** Primary progressive aphasia, Dementia, Visual rating scales, Atrophy, Biomarkers, Magnetic resonance imaging

## Abstract

**Introduction:**

Differential diagnosis among subjects with Primary Progressive Aphasia (PPA) can be challenging. Structural MRI can support the clinical profile. Visual rating scales are a simple and reliable tool to assess brain atrophy in the clinical setting.

The aims of the study were to establish to what extent the visual rating scales could be useful in the differential diagnosis of PPA, to compare the clinical diagnostic impressions derived from routine MRI interpretations with those obtained using the visual rating scale and to correlate results of the scales in a voxel-based morphometry (VBM) analysis.

**Method:**

Patients diagnosed with primary progressive aphasia (PPA) according to current criteria from two centers—Ospedale Maggiore Policlinico of Milan and Hospital Clínic de Barcelona—were included in the study. Two blinded clinicians evaluated the subjects MRIs for cortical atrophy and white matter hyperintensities using two protocols: routine readings and the visual rating scale. The diagnostic accuracy between patients and controls and within PPA subgroups were compared between the two protocols.

**Results:**

One hundred fifty Subjects were studied. All the scales showed a good to excellent intra and inter-rater agreement. The left anterior temporal scale could differentiate between semantic PPA and all other variants.

The rater impression after the protocol can increase the accuracy just for the logopenic PPA. In the VBM analysis, the scores of visual rating scales correlate with the corresponding area of brain atrophy.

**Conclusion:**

The Left anterior temporal rating scale can distinguish semantic PPA from other variants. The rater impression after structured view improved the diagnostic accuracy of logopenic PPA compared to normal readings. The unstructured view of the MRI was reliable for identifying semantic PPA and controls. Neither the structured nor the unstructured view could identify the nonfluent and undetermined variants.

## Introduction

Primary progressive aphasias (PPA) are a group of neurodegenerative conditions characterized by progressive degeneration of the language. The current criteria recognize three variants of PPA: semantic variant PPA (svPPA), characterized by gradual deterioration of semantic representations manifesting as deficits in single-word comprehension and expression; logopenic variant PPA (lvPAA), characterized by deficits in phonological short-term memory resulting in difficulty with naming and repetition, especially for multisyllabic words and sentences; and nonfluent agrammatic variant PPA (nfvPPA), characterized by effortful and poorly articulated speech output with impaired syntactic production and comprehension [8]. However, some cases do not fulfil the criteria labelled PPA undetermined (uPPA) [20]. The differential diagnosis between the variants can sometimes be challenging, but it is of importance for differences in treatment [21].

The diagnosis of PPA variants is challenging due to the overlap of clinical phenotypes. The clinical classification is supported by imaging showing specific patterns of atrophy at CT or MRI: nfvPPA with predominant left posterior fronto-insular atrophy, svPPA predominant anterior temporal lobe atrophy while predominant left posterior perisylvian or parietal atrophy for lvPPA. FDG-PET hypometabolism/SPECT hypoperfusion pattern might also support the clinical diagnosis, but their availability is still limited.

Previous studies applied sophisticated data-driven approaches to characterize atrophy, but these methods may be difficult to replicate in the clinical setting. On the contrary, visual rating atrophy scales represent accessible and reliable measures of cerebral atrophy.

Visual rating scales have proven to provide a reliable, inexpensive, quick and easy-to-assess method in the differential diagnosis of degenerative dementia, such as genetic forms of Frontotemporal dementia or clinical variants of Alzheimer’s disease [3, 5, 6, 10].

## Objectives

The objective of the study was to establish to what extent the visual rating scales could be useful in the differential diagnosis among the different PPAs and which scale is better for each comparison.

The secondary objective was to determine if a structured view for reviewing MRI can increase the accuracy of the diagnosis by the clinician.

Thirdly, we wanted to explore the relationship between the scores of each rating scale with the volume of gray matter using a voxel-based method.

## Methods

### Subjects

Participants were retrospectively recruited at 2 different centres: the Neurodegenerative Diseases Unit of the Fondazione Ca’ Granda, IRCCS Ospedale Maggiore Policlinico, Milan, Italy from June 2012 to August 2019 and Alzheimer’s Disease and other Cognitive Disorders unit of the Hospital Clínic de Barcelona, Barcelona, Spain, from October 2005 to August 2019. All the subjects with a diagnosis of PPA according to current criteria [8] that underwent MRI were included. All participants have provided informed written consent to participate in clinical research.

All the subjects underwent a general and neurological examination, detailed clinical history, comprehensive neuropsychological evaluation, and structural brain imaging. When clinically indicated functional neuroimaging (with FDG-PET or SPECT) and amyloid biomarkers (with CSF or Amyloid-PET) were performed.

Exclusion criteria for this study included aphasia due to stroke or vascular origin or substantial MRI T2 white matter hyperintensities.

### MRI

- MRI acquisition Milan
  The MRI was performed with a 3 Tesla scanner (Achieva, Philips Healthcare, Eindhoven, Netherlands) using a 32-channel phase-array head coil. Whole-brain tridimensional (3D) T1-weighted turbo field-echo sequence was acquired in the sagittal plane. For clinical purposes the MRI protocol also included 3D T2-weighted Fluid Attenuated Inversion Recovery (FLAIR) images, axial fast spin-echo T2-weighted images and axial diffusion-weighted.
- MRI acquisition Barcelona
  High-resolution T1-weighted images were acquired in a 3 Tesla scan (Siemens Magnetom Trio, Erlangen, Germany) at the Magnetic Resonance Image Core Facility, using proprietary three-dimensional magnetization-prepared rapid acquisition gradient echo.
- Visual rating protocol
  A protocol of visual rating scales of atrophy, as described in previously published papers [5, 6, 9], was applied independently by two raters (GF and NF, both neurologists with previous experience with visual rating in dementia) blind for all the demographic and clinical information. In particular, the scales used in the protocol were: Orbitofrontal (OF), Anterior cingulate (AC), Anterior Temporal (AT), Fronto-insula (FI), Medial Temporal (MTA) and Posterior scale (PA).
  Briefly, OF and AC scales, that evaluate respectively olfactory sulcus and cingulate sulcus, are rated in the coronal plane on the most anterior slice where the corpus callosum becomes visible with a four-part grading system: grade 0, representing no atrophy (no cerebrospinal fluid [CSF] visible within the sulcus); grade 1, mild widening of the sulcus (CSF just becomes visible); grade 2, moderate widening; and grade 3, severe widening (with the sulcus assuming a triangular shape). The AT scale looked at the aspects of the temporal pole in coronal view, using a 5-point system: grade 0 representing normal appearances, grade 1 only slight prominence of anterior temporal sulci, grade 2 definite widening of the temporal sulci, grade 3 severe atrophy and ribbon-like nature of the gyri, and grade 4 a simple linear profile of the temporal pole. The FI scale is a 4 point scale evaluating the circular sulcus of the insula in the coronal view on the slice where the anterior commissure become visible and the two following posterior. The MTA is a 5-point graded scale that looks at the medial temporal lobe in coronal view: grade 0 is normal; grade 1 a widened choroidal fissure; grade 2 an increased widening of the choroidal fissure, widening of temporal horn and opening of other sulci; grade 3 pronounced volume loss of the hippocampus; and grade 4 end-stage atrophy. PA scale is a 4-point scale evaluating posterior cortical atrophy using three views (coronal, axial and sagittal): grade 0 representing closed posterior cingulate and parieto-occipital sulci; grade 1 mild widening of the posterior cingulate and parieto-occipital sulci, with mild atrophy of the parietal lobes and precuneus; grade 2 substantial widening of the posterior cingulate and parieto occipital sulcus, with substantial atrophy of the parietal lobes and precuneus; and grade 3 end-stage atrophy with evident widening of both sulci and knife-blade atrophy of the parietal lobes and precuneus. Furthermore, the Posterior scale has been divided into four subscales, one evaluated in the coronal view (Dorsal Parietal (DP) and three in the sagittal view posterior cingulate (PCS), precuneus (PRE) and parieto-occipital (POS). The right and left sides were rated separately for each scale.

To evaluate white matter changes, the protocol included also the modified Fazekas scale [4, 22]. In the Fazekas scale, the degree of white matter changes is rated on a 4-point scale as periventricular WMCs (FAZ PV) and deep white matter hyperintensities (FAZ WMH) in an axial T2-weighted or T2 FLAIR image.

Grade 0 has no or occasional punctate white matter changes and grade 1 has multiple punctate white matter changes. Grade 2 implies incipient confluence or bridging of punctate changes and grade 3 consists of confluent white matter changes.

**Fig. 1:**
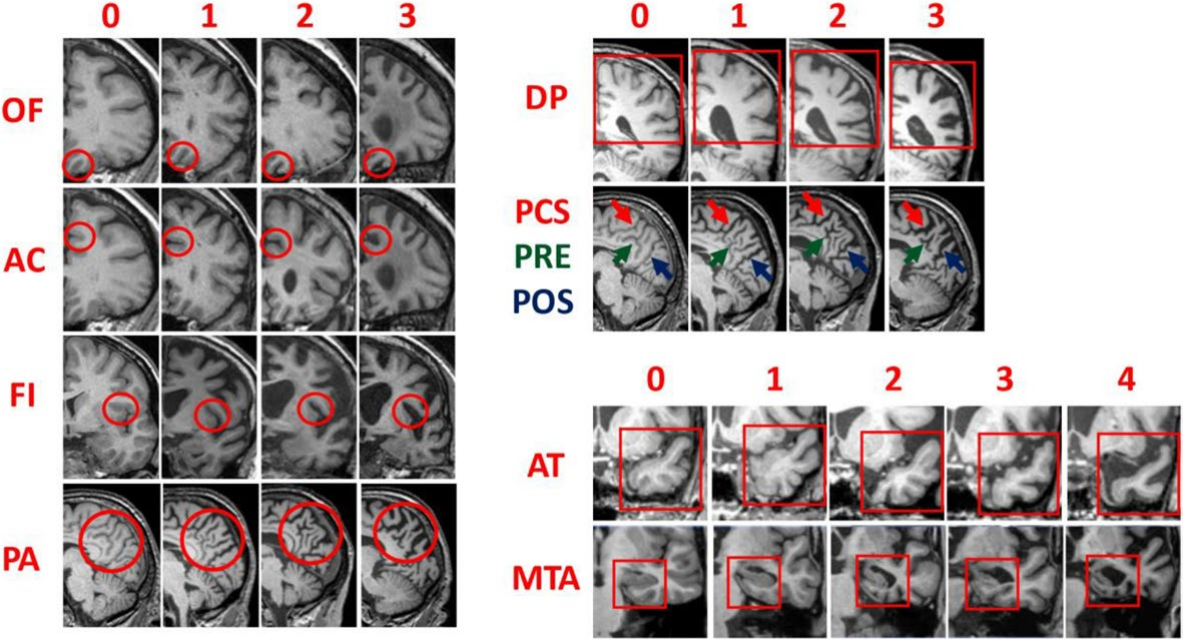
Reference images for visual rating scales of atrophy. Examples of visual rating scores from 0 to 3 for orbitofrontal (OF), anterior cingulate (AC), fronto-insula (FI), posterior (PA), dorso-parietal (DP), posterior cingulate sulcus (PCS), precuneus (PRE), parieto-occipital sulcus (POS) and from 0 to 4 for anterior temporal (AT) and medial temporal (MTA)

**Fig. 2:**
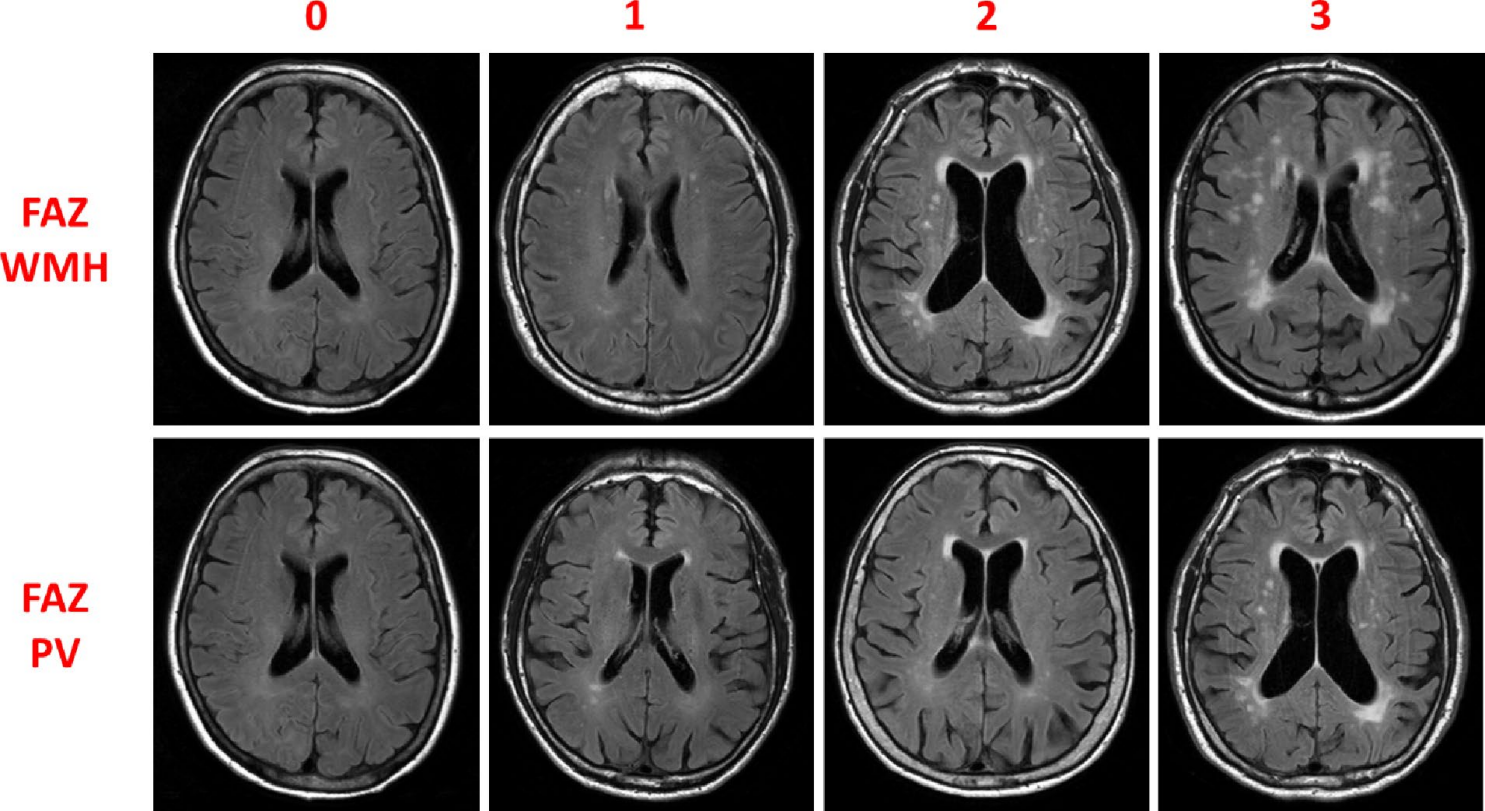
Reference images for visual rating scales of white matter changes. Examples of visual rating scores from 0 to 3 for Fazekas deep white matter hyperintensities (FAZ WMH) and periventricular (FAZ PV) scales

To increase rating consistency, reference images for each scale were provided (Figs. 1 and 2).

Raters were asked to choose one of 5 possible diagnoses (control, lvPAA, svPPA, nfvPPA, uPPA) in an unstructured view and after the visual rating protocol that was done sequentially for each subject. The images were presented in random order.

Lastly, the raters re-rated a subset of 30 randomly chosen subjects to calculate intra-rater reliability. The software used to display images was MRIcron [18]; images have been rated in the native space, in keeping with standard clinical reads.

- Statistics

The program used for the statistical analysis was Jamovi (https://www.jamovi.org/).

Group differences has been tested using t test for age, MMSE and neuropsychological tests, chi squared for gender and ANOVA for visual ratings as they failed Shapiro-Wilk test for normal distribution. Area under the Receiver operating characteristic curve (AUC) was calculated for each significant comparison. For intra and inter-rater agreement weighted Kappa has been calculated. The correlations were analysed with Spearmann rank correlation. Values with *p* < 0.05 were considered statistically significant.

- Voxel based morphometry

VBM analysis was performed using Statistical Parametric Mapping 12 (http://www.fil.ion.ucl.ac.uk/spm). T1- weighted images were normalized and segmented into gray matter (GM), white matter (WM) and CSF probability maps using standard procedures and the fast-diffeomorphic image registration (DARTEL) algorithm [1]. GM segments were affine-transformed into the Montreal Neurological Institute space, then, before the analysis, modulated and smoothed using a Gaussian kernel with 6-mm full-width half-maximum. In order to identify potential outliers, final smoothed-modulated-warped GM images were checked for sample homogeneity using CAT12 toolbox. The GM tissue maps were fitted to a multiple regression model with the aim of identifying correlations with the visual rating scales. Age, gender, total intracranial volume and centre were entered as covariates. Group comparison was made on a voxel-level using two-sample t-tests. To highlight only areas that could have clinical utility the significance threshold was set at 0.05 corrected for multiple comparison (family-wise error) when comparing groups of patients with controls and at 0.001 cluster level corrected when comparing between groups of PPA.

## Results

### Demographic

Demographical data are shown in Table 1. A total of 150 subjects were recruited in the study. 105 had a diagnosis of PPA: Forty-four had a diagnosis of lvPPA, 19 of nfPPA, 31 svPPA and 11 uPPA. In 76 patients functional neuroimaging (either with PET-FDG or SPECT) was performed while 92 patients had their Amyloid status tested.

A total of 45 age and gender-matched controls (HC) without cognitive deficits were recruited for the study (30 Barcelona, 15 Milan). Genetic mutations were found in 11 patients: 6 mutations in the *GRN* gene (5 uPPA and 1 lvPPA), 2 with *C9orf72* expansion (1 lvPPA and 1 svPPA), 1 in *MAPT* (svPPA), 1 PSEN1 (svPPA) and 1 APP (svPPA).

The groups were comparable in terms of age except for the comparison between lvPPA and HC and gender except for the comparison between svPPA and uPPA. MMSE was significantly higher for controls compared to all PPA subtypes. svPPA had a significantly higher MMSE compared to lvPPA and nfPPA. No difference was found in the comparison of the other groups.

**Table 1:** Demographic data of the sample

Table 1	Logopenic	Nonfluent	Semantic	Undetermined	Controls	Significance
**Number**	44	19	31	11	45	
**Age Mean(SD)**	69.8 (7.7)	69.2 (7.6)	66.6 (8.5)	64.5 (11.2)	66.1 (8.6)	*logopenic and controls
**Time since onset in years** **Mean (SD)**	2.87 (1.78)	3.63 (2.18)	3.11 (2.39)	2.28 (1.38)		
**Gender**	25 F 19 M	10 F 9 M	11 F 20 M	8 F 3 M	23 F 22 M	*semantic and undetermined
**MMSE**	20.6	20.6	25.1	22.3	28.8	*controls and all other, logopenic and semantic, semantic and nonfluent

*p <0.05

### Unstructured and structured view

**Fig. 3:**
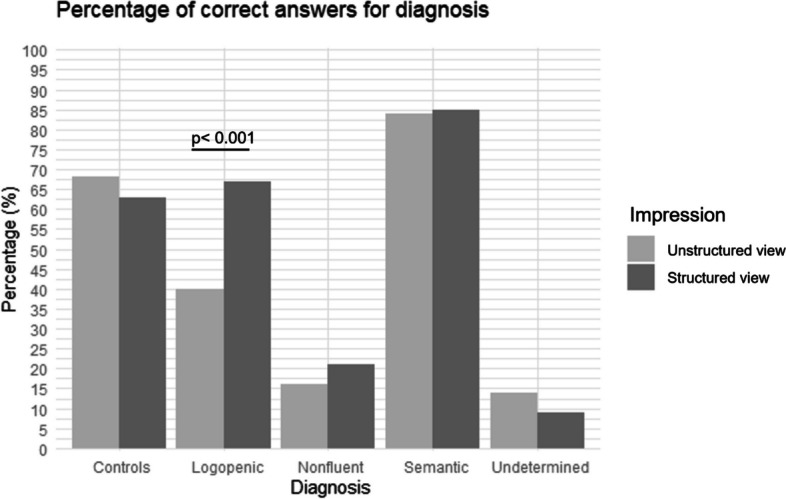
Percentage of correct answers for diagnosis with the unstructured and structured view

The raters could identify correctly in the unstructured assessment 84% of svPPA and 68% of Controls but only 14% of undetermined and 16% of nfvPPA (see Fig. 3). Regarding lvPPA in the unstructured view the raters guessed correctly 40% of subjects, while after the structured view, the percentage of correct answers increased significantly to 67%. No significant change after the structured view was seen in the other groups.

### Visual rating scales

#### Intra and inter rater

All scales demonstrated good to excellent inter-rater reliability with a weighted Kappa score higher than 0.7 except for FIR 0.66, with ACL and ATL scales performing best overall (Table 2). Considering the intra-rater scores, rater 1 weighted Kappa were greater than 0.78 (FAZ WMH) for all the scales, and rater 2 had weighted Kappa scores greater than 0.75 (DPR).

**Table 2:** Intra and Inter-rater agreement

Table 2	Inter rater 1–2	Intra rater 1	Intra rater 2
**OFR**	0.76	0.80	0.84
**OFL**	0.80	0.83	0.84
**ACR**	0.71	0.88	0.86
**ACL**	0.93	0.94	0.95
**FIR**	0.66	0.82	0.87
**FIL**	0.78	0.90	0.92
**ATR**	0.81	0.95	0.94
**ATL**	0.93	0.94	0.95
**MTAR**	0.70	0.91	0.91
**MTAL**	0.86	0.91	0.95
**PAR**	0.73	0.81	0.88
**PAL**	0.70	0.82	0.82
**DPR**	0.73	0.85	0.75
**DPL**	0.78	0.85	0.86
**PCSR**	0.83	0.84	0.89
**PCSL**	0.79	0.90	0.89
**PRER**	0.78	0.79	0.80
**PREL**	0.80	0.81	0.83
**POSR**	0.81	0.85	0.90
**POSL**	0.83	0.89	0.86
**Faz PV**	0.79	0.87	0.76
**Faz WMH**	0.79	0.78	0.86

*OF* Orbitofrontal, *AC* Anterior cingulate, *FI* Fronto-insula, *AT* Anterior temporal, *MTA* Medial temporal, *PA* Posterior, *DP* Dorso-parietal, *PCS* Posterior cingulate sulcus, *PRE* Precuneus, *POS* Parieto-occipital sulcus, *FAZ PV* Fazekas periventricular, *FAZ WMH* Fazekas white matter hyperintensities. *R* Right, *L* Left

#### Mean scores

Detailed rating scores per group are summarized in Table 3.

**Table 3:** Mean scores and standard deviation of each scale for each group

Scale	Side	lvPPA	nfvPPA	svPPA	uPPA	HC	lvPPA - HC	nfvPPA - HC	svPPA - HC	uPPA - HC	lvPPA - nfvPPA	lvPPA - svPPA	lvPPA - uPPA	nfvPPA - svPPA	nfvPPA - uPPA	svPPA - uPPA
**OF**	**R**	0.66 ± 0.72	0.63 ± 0.81	0.87 ± 0.75	1.00 ± 0.71	0.17 ± 0.35	**		***	**						
	**L**	0.83 ± 0.75	0.71 ± 0.92	1.02 ± 0.79	1.55 ± 1.01	0.14 ± 0.29	***	*	***	***						
**AC**	**R**	1.00 ± 0.66	1.03 ± 0.94	0.95 ± 0.51	1.45 ± 0.69	0.48 ± 0.57	*		**	***						
	**L**	0.88 ± 0.70	1.24 ± 0.93	1.18 ± 0.77	1.55 ± 0.88	0.50 ± 0.55		*	**	**						
**FI**	**R**	1.33 ± 0.76	1.42 ± 0.82	1.48 ± 0.64	1.18 ± 0.51	0.78 ± 0.55	*	*	***							
	**L**	1.73 ± 0.77	1.63 ± 0.66	2.08 ± 0.63	2.36 ± 0.92	0.80 ± 0.56	***	***	***	***						
**AT**	**R**	1.11 ± 0.76	0.84 ± 0.99	2.15 ± 1.18	1.09 ± 0.94	0.40 ± 0.50	***		***			***		**		
	**L**	1.72 ± 0.80	1.32 ± 1.15	3.50 ± 0.68	2.00 ± 1.05	0.34 ± 0.50	***	***	***	***		***		***		**
**MTA**	**R**	0.73 ± 0.69	0.45 ± 0.52	1.26 ± 0.97	0.64 ± 0.64	0.22 ± 0.38	***		***					*		
	**L**	1.10 ± 0.88	0.82 ± 0.73	2.27 ± 0.95	1.27 ± 0.88	0.19 ± 0.42	***	**	***	***		***		***		*
**PA**	**R**	1.68 ± 0.82	1.47 ± 0.87	1.24 ± 0.85	1.05 ± 0.57	0.68 ± 0.72	***	**	*							
	**L**	2.01 ± 0.77	1.84 ± 0.83	1.48 ± 0.82	1.55 ± 0.47	0.83 ± 0.70	***	***	**	*		*				
**DP**	**R**	1.73 ± 0.79	1.63 ± 0.83	1.40 ± 0.81	1.27 ± 0.65	0.81 ± 0.81	***	**	*							
	**L**	2.01 ± 0.77	1.84 ± 0.83	1.73 ± 0.83	1.86 ± 0.71	0.94 ± 0.81	***	**	**	*						
**PCS**	**R**	1.76 ± 1.07	1.79 ± 1.03	1.53 ± 0.89	1.27 ± 0.82	1.10 ± 0.86	*									
	**L**	1.36 ± 0.89	1.03 ± 0.82	0.97 ± 0.90	0.59 ± 0.58	0.54 ± 0.61	***									
**PRE**	**R**	1.43 ± 0.91	1.47 ± 0.99	1.05 ± 0.86	0.91 ± 0.70	0.70 ± 0.81	**	*								
	**L**	1.99 ± 1.03	1.97 ± 0.86	1.69 ± 0.84	1.64 ± 0.64	1.20 ± 0.76	**	*								
**POS**	**R**	1.63 ± 0.95	1.34 ± 1.13	1.26 ± 0.95	1.05 ± 0.47	0.54 ± 0.52	***		**							
	**L**	1.74 ± 0.94	1.53 ± 1.03	1.29 ± 0.91	1.32 ± 0.60	0.72 ± 0.82	***	*	*							
**Faz PV**		1.16 ± 0.91	0.81 ± 0.49	0.87 ± 0.81	0.59 ± 0.97	0.50 ± 0.60	***									
**Faz WMH**		1.02 ± 0.85	0.67 ± 0.59	0.75 ± 0.79	0.59 ± 0.77	0.59 ± 0.59										

*OF* Orbitofrontal, *AC* Anterior cingulate, *FI* Fronto-insula, *AT* Anterior temporal, *MTA* Medial temporal, *PA* Posterior, *DP* Dorso-parietal, *PCS* Posterior cingulate sulcus, *PRE* Precuneus, *POS* Parieto-occipital sulcus, *FAZ PV* Fazekas periventricular, *FAZ WMH* Fazekas white matter hyperintensities. *R* Right, *L* Left. *lvPPA* Logopenic, *nfvPPA* Nonfluent, *svPPA* Semantic, *uPPA* Undetermined, *HC* Controls. *LOG* Logopenic, *NFL*Nonfluent, *SEM* Semantic, *UND* Undetermined, *CON* Controls

*p < 0.05 **p < 0.01 ***p < 0.001

- Group comparisons
  - ◦ **PPA subgroups to controls comparison**
    - Logopenic vs. controls
    - The scores of all the visual rating scales except for ACL and FAZ WMH were significantly higher in logopenic than controls.
    - Nonfluent vs Controls
    - nfvPPA had higher scores than controls in OFL, ACL, ATL, FIR, FIL, MTAL, PAR, PAL, DPR, DPL, PRER, PREL, and POSL.
    - Semantic vs Controls
    - Compared to controls, svPPA had higher scores in all the scales except for the parietal subscales PCS and PRE and the two Fazekas scales.
    - Undetermined vs Controls
    - Undetermined obtained a higher score in OF and AC on both sides and on ATL, FIL, MTAL, PAL and DPL.
    ◦ **Comparison among groups of PPA**
    - No scale showed differences in the comparisons between lvPPA with nfvPPA and with uPPA as well as in the comparison between nfvPPA with uPPA. In the comparison between lvPPA and svPPA, svPPA had higher scores in ATR, ATL and MTAL while lvPPA had higher scores in PAL. svPPA got higher scores in AT and MTA scales compared to nfvPPA while only on ATL and MTAL compared to uPPA.

#### Rating scales diagnostic performance

Detailed diagnostic performance for each group comparison are shown in Table 4.

**Table 4:** ROC curve analysis for significant comparisons. Only the scale with higher AUC is indicated

Table 4	lvPPA-HC	nfvPPA-HC	svPPA-HC	uPPA-HC	svPPA-lvPPA	svPPA- nfvPPA	svPPA-uPPA
**Best Scale**	ATL	PAL	ATL	ATL	ATL	ATL	ATL
**Cutpoint**	1	2	2	1	3	2	3
**Sensitivity (%)**	88.64%	57.89%	100%	100%	83.87%	100%	83.87%
**Specificity (%)**	86.67%	88.89%	97.78%	86.67%	95.45%	73.68%	81.82%
**PPV (%)**	86.67%	68.75%	96.88%	64.71%	92.86%	86.11%	92.86%
**NPV (%)**	88.64%	83.33%	100%	100%	89.36%	100%	64.29%
**AUC**	0.921	0.817	0.999	0.953	0.940	0.925	0.868

*ATL* Anterior temporal left, *PAL* Posterior left, *lvPPA* Logopenic, *nfvPPA* Nonfluent, *svPPA* Semantic, *uPPA* Undetermined, *HC* Controls. *PPV* Positive predictive value, *NPV* Negative predictive value

For each comparison, only the scale that showed the best result was considered.

AUC of ROC curves in the comparison between each group with all the other subjects resulted in values ranging from 0.633 for nfvPPA to 0.953 for svPPA.

Compared to controls ATL was the scale that showed a higher AUC for lvPPA, svPPA and uPPA; for nfvPPA the best scale was PAL.

In the direct comparison between PPA groups, ATL was the best scale for comparing svPPA with lvPPA, nfvPPA and uPPA.

#### VBM group differences

**Fig. 4:**
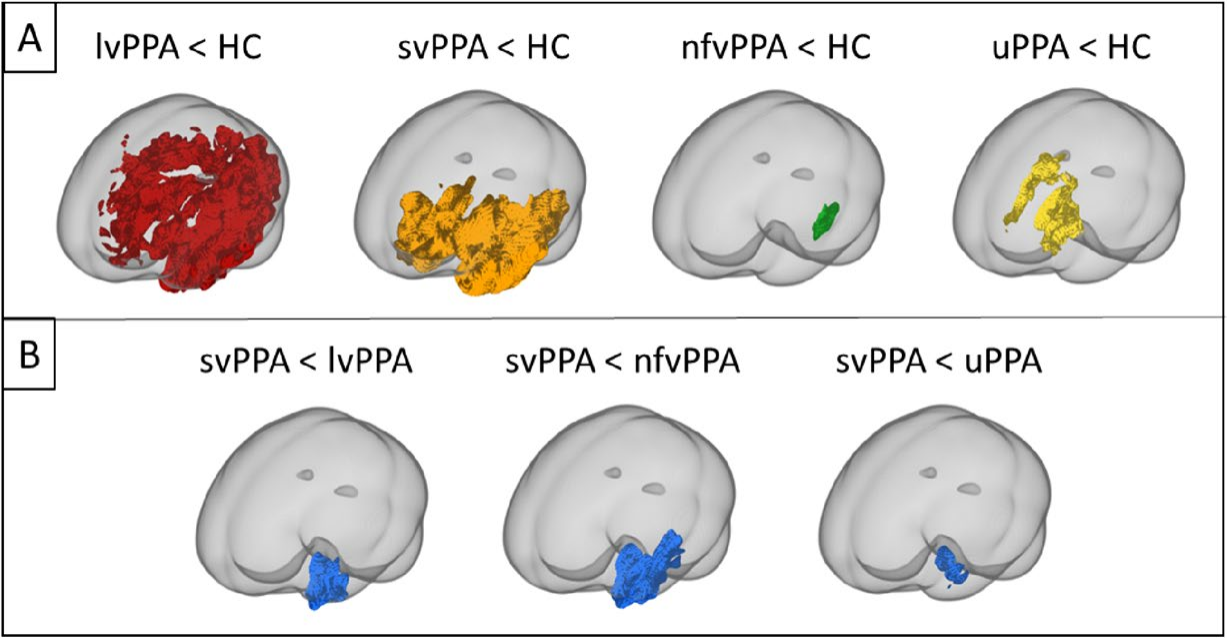
Results of voxel-based morphometry analysis of the difference between groups Box **A** comparison of each group with healthy controls. Box **B** comparison between semantic PPA with each other group. lvPPA logopenic, nfvPPA nonfluent, svPPA semantic, uPPA undetermined, HC controls

Compared to controls, the svPPA group was characterized by atrophy in the anterior temporal lobes, nfvPPA by atrophy in left posterior frontal and insula, lvPPA by left posterior temporoparietal atrophy, while uPPA left frontal lobe and caudate nucleus (see Fig. 4A).

Between PPA groups comparison revealed that svPPA had an area of greater atrophy in the left anterior temporal lobe compared to the other three groups (see Fig. 4B).

### VBM correlations with visual rating scales

**Fig. 5:**
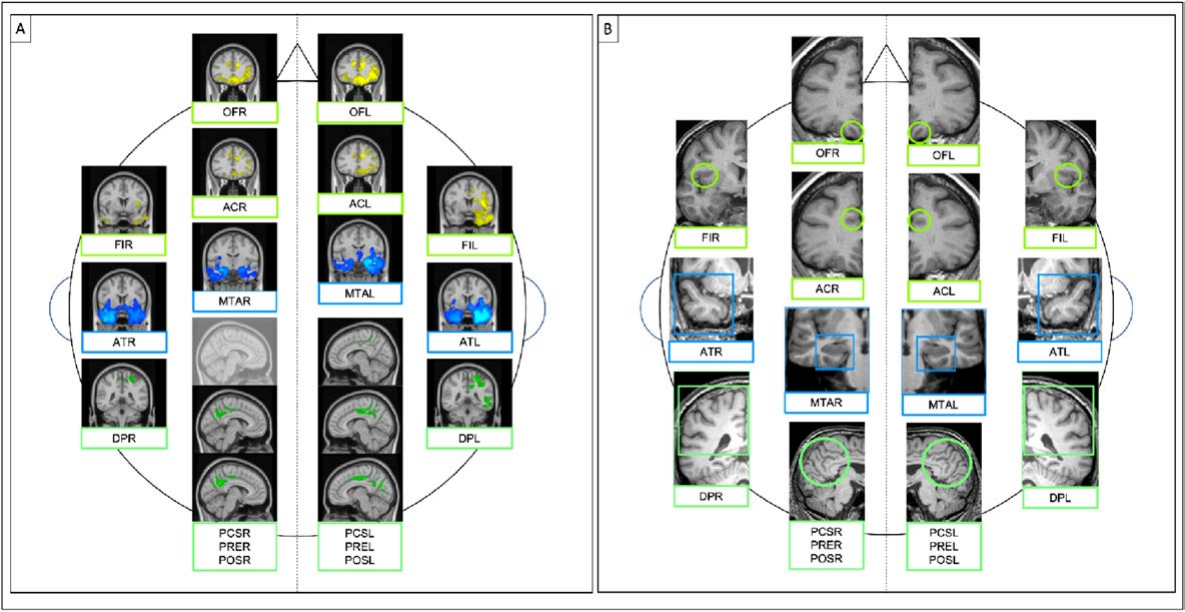
Box **A** results of VBM correlation analysis between GM and visual rating scores for each scale Box **B** area of interest for each visual rating scale OF orbitofrontal, AC anterior cingulate, FI fronto-insula, AT anterior temporal, MTA medial temporal, PA posterior, DP dorso-parietal, PCS posterior cingulate sulcus, PRE precuneus, POS parieto-occipital sulcus, R right, L left

The analysis revealed an inverse correlation of the scores from each visual rating scale with an area of GM atrophy in the same expected region except for PCSR (Fig. 5).

## Discussion

In this study, we investigated the utility of visual rating scales in the differential diagnosis of PPA for a clinical application.

We found that the single left anterior temporal scale should be the scale of choice to confirm or exclude patients with semantic dementia. In our study, the left anterior temporal scale has proven to be the most useful to differentiate PPA from controls, but also for the subdivision of PPA. The highest levels of atrophy were, as expected, among semantics.

These results are in line with previous studies on the topic. Particularly, Sajjadi et al. [19] used a non-structured visual classification from radiological reports to the question, proposing imaging markers for each variant and extracting them from neuroradiologist reports (temporal lobe atrophy for svPPA; left frontal or left frontotemporal atrophy for nfvPPA; left temporal, left parietal, or left temporoparietal atrophy for lvPPA). They found a high sensitivity and specificity for svPPA, but less reliable outcomes for lvPPA and nfvPPA, with a low sensitivity but relatively high specificity [19]. The visual assessment was, however based on neuroradiological reports and not on structured visual rating, and our approach showed a higher inter-rater agreement.

We could not differentiate between nfvPPA and lvPPA, but these results are in line with previous works using structural volumetric analysis such as VBM or cortical thickness [2, 23]. Between these two variants the diagnosis should rely more on the clinical and neuropsychological profile, rather than structural imaging.

Regarding white matter changes, only the visual rating scale of periventricular hyperintensities found a difference between lvPPA and HC. The Fazekas scale has shown to be correlated with poor naming and sentence repetition in a cohort of PPA [15], but the authors did not test for differences between groups of PPA.

One of the main findings of the study was the increase in the accuracy of the diagnosis of lvPPA after the structured analysis. The unstructured view of the MRI can be reliable for identifying svPPA and controls but not for the nfvPPA and uPPA. These results do not change after the structured analysis. We can assume that an unstructured view can give enough strength to confirm or exclude a semantic or a control, but with a visual rating of the left anterior temporal, this confidence can increase.

Both the raters were experts with previous experience in visual rating but the structured view may also help non experts focus on relevant areas. The other side of the coin is that the structured view did not help to increase the accuracy of the other variants.

Recently Pemberton et al. reported that using quantitative reports alongside routine visual MRI assessment improved sensitivity and accuracy to discriminate Alzheimer’s disease from Frontotemporal dementia compared to visual assessment alone [17]. In this context and considering the difference in accuracy between the visual rating and the raters’s impressions, the practice of adding quantitative scores to the report in addition to the visual assessment would be advisable. In real life clinical settings, the diagnostic performance of visual rating scales has shown similar results to automated volumetric quantification, which is not feasible in up to 30% of the cases [11, 13].

Bisenius et al. analysed structural MRI of PPA using a support vector machine and found that this method was able to discriminate with high accuracy PPA subtypes from healthy controls and also svPPA from lvPPA and NFL, but the accuracy between lvPPA and nfvPPA was low. In our study, we found comparable results of positive and negative predictive values with their approach to regions of interest, but using a more straightforward and cheaper method that could be applied in the clinic [2].

More recently, Manouvelou et al. described that a combination of different visual rating scales performed better than single scales in the comparison scales between svPPA and bvFTD [14].

The group-level VBM GM atrophy patterns for each of the PPA variants were consistent with those in previous studies with left posterior fronto-insular atrophy in nfvPPA, anterior temporal atrophy more pronounced on the left in svPPA and predominant left temporoparietal atrophy in lvPPA [7, 12, 16]. The rating scores obtained for each PPA group overall meet the characteristic pattern of atrophy therefore, the visual rating scores can give comparable results reinforcing their relevance from a clinical point of view.

The VBM correlation analysis confirmed that the area of major correlation for each visual rating scale corresponds to the expected area in validation using an unbiased approach, as has already been shown [5, 6, 9]. This validation, together with the good intra and inter -rater agreement values, is relevant because it shows the visual rating method’s ability to provide reliable results despite being less sophisticated. Conversely, the visual rating scales are more applicable in an outpatient setting.

The study was intended to resemble the clinical practice, however, in the clinics, the physician has more information regarding the patient such as age, symptoms, or disease duration. These pieces of information can potentially increase diagnostic accuracy; in fact, models with both imaging and linguistic features performed better than models with only imaging and only linguistic features [23]. However, given that the first clinical impression of the clinician may influence their approach in assessing/interpreting the MRI, for this study we preferred to keep the analysis bias-free. One of the strengths was to have studied an elevated number of cases, even though from two different centres.

This study has several limitations. A limitation is related to the rating scales themselves. Visual, qualitative scales are subjective, gross measures of brain atrophy, however, in the present study, inter and intra-rater reliability were good to excellent for all the scales. The retrospective nature of the study based on data collected in the routine clinical practice, is another limitation due to the lack of harmonization of neuropsychological measures and is also related to the different languages used in the two centres. In the end, the neuropathological confirmation of the diagnosis is lacking, but the cohort has been well characterized from a clinical and biomarker-based perspective.

## Conclusions

A structured observation of the MRI with visual rating scales can increase the diagnostic accuracy for lvPPA. Unstructured expert review is sufficient to confirm or exclude svPPA from other PPAs.

## Data Availability

The data supporting the findings of this study are available upon reasonable request from the corresponding author.

## Abbreviations

AC: Anterior cingulate rating scale
AT: Anterior Temporal rating scale
AUC: Area under the Receiver operating characteristic curve
CSF: Cerebrospinal fluid
DP: Dorsal Parietal rating scale
FAZ PV: Fazekas periventricular rating scale
FAZ WMH: Fazekas deep white matter hyperintensities rating scale
FI: Fronto-insula rating scale
GM: Gray matter
HC: Healty controls
lvPAA: logopenic variant primary progressive aphasia
MMSE: Mini Mental state examination
MTA: Medial Temporal rating scale
nfvPPA: nonfluent/agrammatic variant primary progressive aphasia
OF: Orbitofrontal rating scale
PA: Posterior rating scale
PCS: Posterior cingulate rating scale
POS: Parieto-occipital rating scale
PPA: Primary progressive aphasias
PRE: Precuneus rating scale
svPPA: Semantic variant primary progressive aphasia
uPPA: Undetermined primary progressive aphasia
VBM: Voxel based morphometry
WM: White matter
